# Alzheimer’s Disease and Related Dementias IV A Decade of PET for Amyloid Imaging: Insights from Real-World Evidence Supporting Alzheimer’s Diagnosis

**DOI:** 10.1093/geroni/igaf122.3172

**Published:** 2025-12-31

**Authors:** Margaret Cadden, Samantha Rossano, Brittany Watson, Marianne Chapleau, Santiago Bullich, Andrew Stephens, Norman Koglin, Aleksandar Jovalekic

**Affiliations:** Life Molecular Imaging, Boston, Massachusetts, United States; Life Molecular Imaging, Boston, Massachusetts, United States; Life Molecular Imaging, Boston, Massachusetts, United States; Life Molecular Imaging, Boston, Massachusetts, United States; Life Molecular Imaging, Berlin, Brandenburg, Germany; Life Molecular Imaging, Berlin, Brandenburg, Germany; Life Molecular Imaging, Berlin, Brandenburg, Germany; Life Molecular Imaging, Berlin, Brandenburg, Germany

## Abstract

Positron emission tomography (PET) scanning with florbetaben (FBB) was approved in the US in 2014 after validation in histopathological studies for imaging of amyloid plaques in individuals being evaluated for Alzheimer’s disease (AD). The current abstract incorporates real-world evidence of FBB’s clinical and research use, including results from visual and quantitative assessment of amyloid PET images. The objectives of this work included 1) Evaluating the efficacy of the approved visual assessment method in the routine clinical practice and 2) Presenting results of 15 quantitative pipelines to underscore reliability and added value of quantification. The studies’ results confirmed that, both experts and naïve readers accurately assess FBB PET scans and maintain these abilities over time (correct assessments: 96% at baseline and 92% at 6 months follow-up). Quantitative methods, including 510(k) approved software, consistently demonstrated robust and homogeneous performance (accuracy 96.4±1.1%), offering valuable support for visual assessments. Since its approval, visual assessment and quantification of FBB images have undergone additional validation across diverse settings including clinical routine. FBB PET imaging is a validated, reliable, and available tool in the diagnostic pathway of AD, as indicated within the Alzheimer’s Association’s (AA) Updated Criteria for AD Diagnosis and Staging and the AA/Society for Nuclear Medicine’s Appropriate Use Criteria for PET. With the approval of amyloid-targeting therapies, standardized quantification approaches, such as the Centiloid scale, are becoming increasingly important for patient selection and treatment monitoring.

